# Mesoporous Silica Nanoparticles for Drug Delivery: Current Insights

**DOI:** 10.3390/molecules23010047

**Published:** 2017-12-25

**Authors:** María Vallet-Regí, Montserrat Colilla, Isabel Izquierdo-Barba, Miguel Manzano

**Affiliations:** 1 Departamento de Química Inorgánica y Bioinorgánica, Facultad de Farmacia, Universidad Complutense de Madrid, Instituto de Investigación Sanitaria Hospital 12 de Octubre i+12, Plaza Ramón y Cajal s/n, 28040 Madrid, Spain; mcolilla@ucm.es (M.C.); ibarba@ucm.es (I.I.-B.); mmanzano@ucm.es (M.M.); 2 Centro de Investigación Biomédica en Red de Bioingeniería, Biomateriales y Nanomedicina (CIBER-BBN), 28040 Madrid, Spain

**Keywords:** mesoporous silica nanoparticles, multifunctional nanosystem, selective targeting, stimuli-responsive drug delivery, in vitro degradation, benefits and downsides, clinical translation, biosafety

## Abstract

This manuscript reviews the recent progress on mesoporous silica nanoparticles as drug delivery systems. Their intrinsic structural, textural and chemical features permit to design versatile multifunctional nanosystems with the capability to target the diseased tissue and release the cargo on demand upon exposition to internal or external stimuli. The degradation rate of these nanocarriers in diverse physiological fluids is overviewed obeying their significance for their potential translation towards clinical applications. To conclude, the balance between the benefits and downsides of this revolutionary nanotechnological tool is also discussed.

## 1. Introduction

Mesoporous silica nanoparticles (MSNs) have revolutionized the field of controlled drug delivery systems. Their advantageous features as well-ordered internal mesopores (typically ca. 2–6 nm) with large pore volume (0.6–1 cm^3^/g) and surface area (700–1000 m^2^/g), tunable size (50–200 nm) and shape, robustness and easy surface modification, make them ideal platforms to design multifunctional nanosystems [1,2,3,4,5]. 

**Figure 1:**
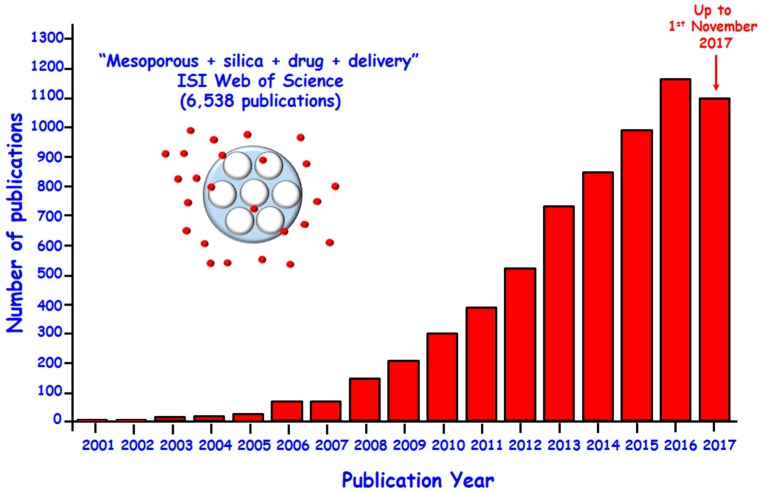
Number of publications per year indexed in the ISI Web of Science on the topic of “mesoporous” and “silica” and “drug” and “delivery” up to 1st November 2017.

Since 2001, when Vallet-Regí et al. [6] introduced for the first time MCM-41 as a drug delivery system, much effort has been devoted to the design of versatile MSNs for treating diverse pathologies, with special emphasis in cancer treatment (Figure 1) [7,8,9,10,11,12,13,14,15,16]. Their high drug loading capability, the possibility to attain localized and even combined therapy make them promising alternatives to develop advanced nanotherapeutics [17,18,19]. Moreover, the textural properties of MSNs play a key role in the performance of these nanosystems as drug delivery devices [1,20]. Thus, the pore diameter behaves as a size selector for the loading of biologically active molecules within the mesoporous cavities. Furthermore, this parameter regulates the release rate, thus acting as a limiting factor that governs molecules diffusion processes to the physiological environment. On the other hand, the surface area determines the molecules loading capacity of these nanoplatforms, because the higher the contact surface the greater the number of guest molecules incorporated. Besides, the pore volume may also influence the amount of drug loaded when aimed at the total filling of the mesopores by promoting not only matrix-guest interactions but also drug-drug interactions. Finally, MSNs are well tolerated in vitro (at dosages < 100 µg/mL) [21,22,23,24] and in vivo (at dosages < 200 mg/kg) [22]. Furthermore, their good hemocompatibility has been also proved [25,26].

Recently, the combination of MSNs with liposomes has been reported, leading to a unique nanocarriers denoted as “protocells” [7]. This nanosystem exhibits the robustness and high loading and controlled release capability of MSNs with the low toxicity and immunogenicity of liposomes. Protocells open up novel expectation in the drug delivery scenario due to their potential to tackle at once multiple challenges, such as stability, specificity and high loading capacity for diverse cargos [27].

The entering of nanomedicine into this landscape is expected to change the near future of pharmaceutical and biotechnological industries. This novel nanotechnological tools would overcome the major constrains of conventional medicine, as low solubility and stability, narrow therapeutic window, lack of specificity and non-adequate pharmacokinetic profiles and severe side effects of drugs [28]. Although during the last few decades the entering of nanomaterials in medicine has delivered more than 250 products already approved or under different phases of clinical trials, their ultimate clinical application remains a great challenge [29].

Herein, the different approaches developed so far to design MSNs owning specificity to the target (diseased tissue, organ or cell) and stimuli-responsive controlled drug dosage ability are overviewed. Moreover, the degradability of these nanosystems during and after drug delivery is also discussed due to their relevance in the successful translation to the clinical arena. Finally, the benefits and downsides of this groundbreaking nanotechnology are summarized.

## 2. Mesoporous Silica Nanoparticles as Drug Delivery Systems

MSNs exhibit unique characteristics that make them ideal nanocarriers to host, protect and transport drugs to the target site. It is feasible to incorporate targeting agents in the external surface of MSNs to direct them to the unhealthy tissues aimed at increasing specificity and therefore diminishing undesired side effects. Another pivotal challenge is to avoid the premature cargo release before reaching the target. In this sense, the pore entrances of MSNs can be capped by using stimuli-responsive gatekeepers. Thus, the exposure to internal or external stimuli would trigger pore opening and allow cargo departure. Besides, it is possible to design multifunctional MSNs with synergistic therapeutic effects against diseased tissues. In this context, these enhanced dual therapies can be achieved by different strategies, as will be discussed below. Since most of the research effort on MSNs for drug delivery has been committed to cancer therapy, in this section we will mainly focus on this pathology.

### 2.1. Selective Targeting for Localized Therapy

**Figure 2:**
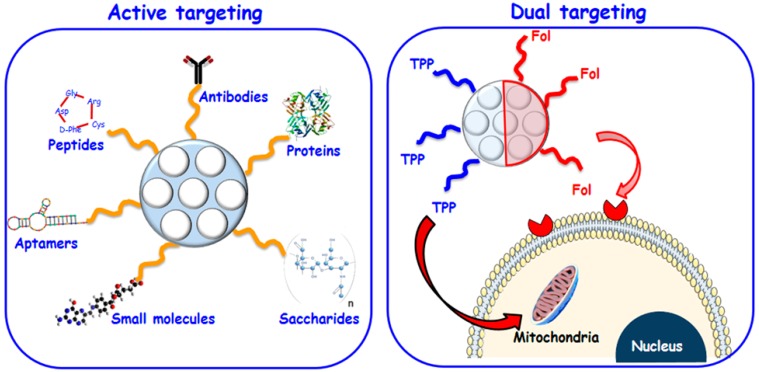
(**Left**) Schematic depiction of active targeting possibilities on MSNs; (**Right**) Dual targeting strategy to target both cell membrane of tumor cells and mitochondria by asymmetrically functionalized nanoparticles (J-MSNs).

In general, nanosystems injected into the blood stream are prompt to accumulate in the tumor zone via the well-known enhanced permeation and retention (EPR) effect due to the peculiar blood vessel architecture of these diseased tissues [30,31]. Nonetheless, the tumor mass is complex and heterogeneous and it is composed by numerous cells (cancerous, supportive and immune cells, etc.) [32]. As an efficient eradication of malignant cells is required, it is essential to provide the nanosystem of capability to discriminate between cancer and healthy cells. A widely explored strategy consists in decorating the outermost surface of MSNs with molecules able to interact selectively with specific membrane receptors overexpressed in tumor cells (Figure 2).

Besides, it is possible to decorate MSNs with targeting ligands with affinity towards the blood vessels that irrigate the solid tumor, which disrupts its nutrients and oxygen supply triggering the tumor destruction. These two approaches, known as active targeting, allow for a noticeable improvement of the particle uptake by the tumor cell or tumor blood vessels [33]. Table 1 summarizes these active targeting strategies developed up to date for MSNs.

On the other hand, it is possible to graft two targeting agents (dual targeting) to the same nanocarrier with the aim of enhancing even more its selectivity [69,70,71]. Thus, the nanocarriers trafficking within the cell can be controlled by placing targeting molecules able to recognize different cell organelles. In a recent study, it has been proved that the best configuration of the targeting agents in dual-targeted nanocarriers is Janus-type structures (J-MSNs) [72]. Very recently, J-MSNs asymmetrically functionalized with targeting agents, folic acid (FA, with affinity towards folate membrane cell receptors overexpressed in cancer cells) and triphenylphospine (TPP, able to bind to mitochondria membrane), has been developed for sequential cell to organelle targeting proposes [46] (Figure 2). The asymmetric functionalization permits a fine control in the targeting grafting process. Hence, the presence of FA increases the accumulation of J-MSNs inside the cancer cells, where they are subsequently driven to mitochondria by TPP action. This dual-targeting strategy can be applied to enhance the therapeutic efficiency of MSNs for antitumor therapies.

One of the major limitations in the use of nanosystems is their poor penetration capability within the tumor mass, due to the presence of a collagen-rich extracellular matrix, which hampers the diffusion of these nanocarriers. Therefore, two main alternatives have been proposed to increase the penetration rate of MSNs into tumor masses. The first one consists in the design of pH-sensitive collagenase nanocapsules anchored on the MSNs surface [73]. Collagenase is a proteolytic enzyme able to digest the extracellular matrix, which improves the penetration degree of the nanosystem and enhances its therapeutic capability. However, because of this enzyme is easily degraded or denaturalized under varied conditions, it has been protected by using nanocapsules formed by radical polymerization of different monomers: acrylamide as the structural monomer; 2-aminoethylmethacrylate to provide amino groups capable of attach to MSNs surface; and ethylene glycol dimethacrylate as the pH-cleavable cross-linker. Thus, under acidic pH typical of solid tumor environment, the nanocapsules break triggering the collagenase release, which then digests the extracellular matrix and improves the penetrability of the MSNs. This novel nanosystem exhibits good biocompatibility, which opens up new paths for further applications in nanomedicine.

The second alternative relies on taking advantage of the capability of human mesenchymal stem cells (MSCs) to migrate towards tumors [74]. Thus, MSCs have been successfully reported as MSNs carriers, being able to reach the deeper regions of diverse tumors [75]. Thus, Paris et al. have designed a new tumor-tropic system consisting in decidua of human placenta MSCs and doxorubicin loaded-MSNs, which induces an efficient cancer cell death both in vitro and in vivo.

### 2.2. Controlled Dosage and Smart Behavior

**Figure 3:**
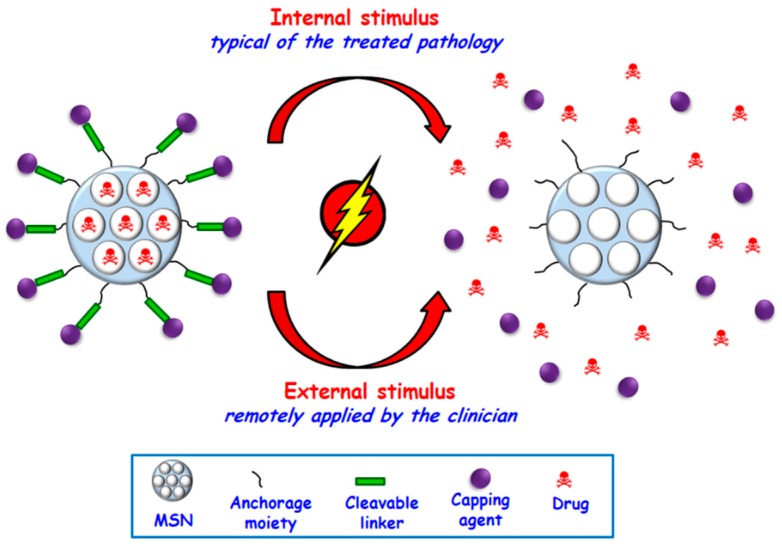
Schematic representation of internal or external stimuli-responsive drug delivery from MSNs.

One of the major advantages of MSNs as drug delivery systems is the possibility to design zero-premature cargo release nanosystems by blocking the pore openings using gatekeepers. Stimuli-responsive behavior can be accomplished anchoring pore blocking caps throughout linkers that can be cleaved upon exposure to given stimuli. These stimuli are classified as internal, i.e., those typical of the treated pathology, such as pH, redox potential, enzymes, etc., and external, such as magnetic fields, ultrasounds or light, among others, which can be remotely applied by the clinician (Figure 3).

Smart MSNs that respond to internal stimuli exhibit the advantage of not needing external devices to trigger cargo release. Nonetheless, the precise control of drug dosage is lower than that achieved using external stimuli. Anyway, each type of smart nanosystem shows pros and cons that should be carefully considered depending on the targeted pathology and their potential clinical application.

#### 2.2.1. Internal Stimuli-Responsive Drug Delivery MSNs

**Figure 4:**
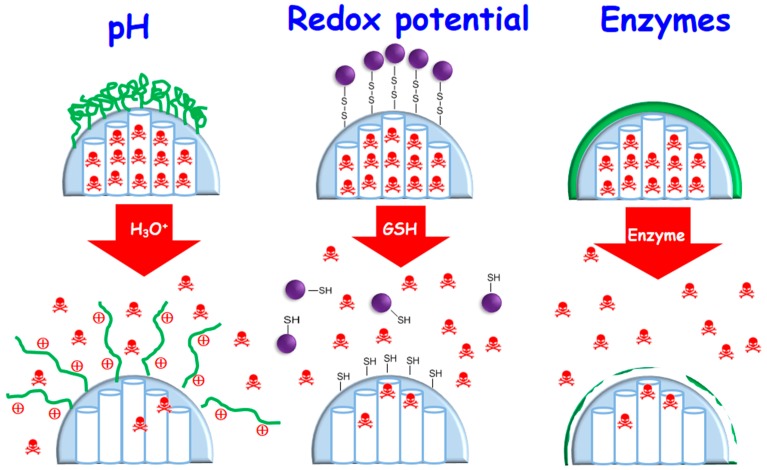
Schematic illustration of representative internal stimuli-responsive drug delivery MSNs: pH-responsive nanosystem based on polymer coated-MSNs; redox potential-responsive based on MSNs functionalized with disulfide bonds and capped with inorganic nanoparticles; and enzyme-responsive based on MSNs coated with a degradable polymer, from left to right.

The deep knowledge of the biochemical and metabolic processes involved in the different pathologies to be treated has allowed for the design of drug delivery nanosystems sensitive to endogenous stimuli [4,76,77]. In this context it has been reported the design of smart MSNs able to respond to specific internal stimuli such as pH variations, high glutathione concentration, overexpression of certain enzymes or presence of several small molecules. In general, these smart drug delivery systems incorporate one or two elements, namely a sensitive linker and/or a capping agent. The responsive linker is able to break, degrade or undergo a conformational change in the presence of the given stimulus. The capping agents, such as inorganic nanoparticles, polymers or macromolecules, block the mesopore entrances and hinders premature cargo departure. It is also possible to use coatings of organic or inorganic chemical nature as blocking caps able to degrade under the stimulus action, thus allowing pore uncapping and drug release (Figure 4). Table 2 summarizes the main internal stimuli-responsive MSNs reported up to date, specifying the endogenous stimulus, the responsive linker and the capping agent.

#### 2.2.2. External Stimuli-Responsive Drug Delivery MSNs

**Figure 5:**
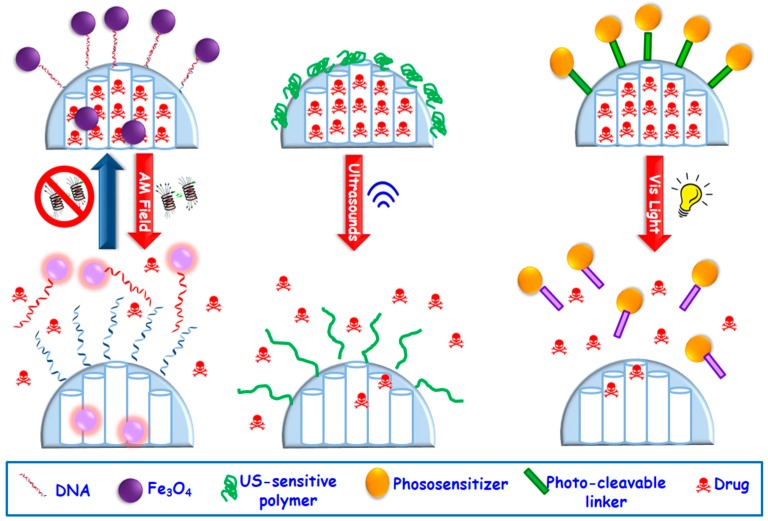
Schematic depiction of three representative external stimuli-responsive drug delivery from MSNs via alternating magnetic fields (AM Field), ultrasounds (US) and visible (Vis) light (from left to right).

Different stimuli-responsive MSNs have been developed capable to respond to externally applied stimuli, highlighting magnetic fields, ultrasound or light, among others (Figure 5) [4,18,76].

- Magnetically-responsive MSNs. The benefits of using magnetic fields is due to the different effect that they can exert on MSNs, which can be magnetic guidance under a permanent magnetic field or a temperature increase upon application of an alternating magnetic (AM) field [76] This permits a wide range of possibilities for in the biomedical field. The most widely used magnetic nanoparticles for stimuli-responsive drug delivery are superparamagnetic iron oxide ones (SPIONs). These nanoparticles are able to convert the magnetic energy into heat obeying two mechanisms: (i) Brownian fluctuations provoked by the fast rotation of the magnetic nuclei, and (ii) Nell fluctuations caused by the rotation of the magnetic moments [101]. Most of the employed designed strategies consist in the encapsulation of SPIONs of ca. 5–10 nm within MSNs, which can be accomplished by using aerosol techniques [102] or sol-gel process [103,104,105,106] etc. The incorporation of SPIONs within MSNs permits the employ of AM fields, which triggers temperature increase. MSNs can incorporate temperature-responsive moieties acting as gatekeepers able to undergo physicochemical changes that provoke pore opening and drug release. Figure 5 displays a representative example based on drug-loaded magnetic MSNs whose pore outlets have been grafted with single-DNA strands that hybridize with Fe_3_O_4_ SPIONs functionalized with the complementary DNA strand, acting as capping agents. The application of an AM field provokes heat that trigger the dehybridization of the DNA, allowing the cargo release in a reversible fashion [107].
- Ultrasound-triggered MSNs. Ultrasounds (US) constitute an efficient method to attain spatiotemporal control of drug delivery at the target site and preventing the damage of healthy tissues. Other of the advantages of the use of US regards its non-invasiveness, absence of ionizing radiations and the easily regulation of tissue penetration depth by tuning US parameters (frequency, duty cycles and exposure times) [76,108]. US waves can trigger drug release from MSNs through thermal effect. Mechanophores, i.e., chemical bonds that cleave under US radiation, can be used to design of US-triggered MSNs. Thus, 2-tetrahydropyranyl methacrylate, a hydrophobic monomer with a US-sensitive group, can transform to hydrophilic methacrylic acid [109,110]. This phase transformation under US stimulus has been used to develop US-responsive drug delivery MSNs by using such moieties as mesopore gatekeepers (Figure 5) [111,112].
- Light-triggered MSNs. Light constitutes another useful alternative with non-invasive and spatiotemporal control to design stimuli-responsive MSNs able to achieve on-demand drug release triggered by illumination with a specific wavelength (ultraviolet, UV, visible, Vis, or near-infrared, NIR, regions) [113,114]. The advantages of the use of light relies on its easy application, low toxicity and precise focalization in the desired place. Nonetheless, the main constrain is its low tissue penetration capability, which can be solved by using auxiliary medical devices such as those use in laparoscopy surgeries. Up to date UV stimulus has been by far the most widely used radiation to trigger drug release from MSNs [36,113] because this light has the highest power and can break bonds with ease. However, UV light present several drawbacks for current biomedical applications, such as its toxicity and low tissue penetrability [115,116,117,118]. Thus, recently Vis light is receiving growing attention since it offers a less harmful and higher penetrability rate than UV radiation. Figure 5 displays a representative example of a Vis light-triggered MSNs-based drug delivery system [119]. In this case, MSNs are decorated with porphyrin nanocaps anchored via reactive oxygen species (ROS)-cleavable linkages. When Vis light stimulus is applied, the porphyrin blocking caps provoke singlet oxygen molecules that break the sensitive linker and trigger the opening of mesopores and allowing drug release.

## 3. Performance in Physiological Fluids

To exploit the potential biomedical usefulness of MSNs as drug nanocarriers, it is essential to understand the final fate of silica matrix in the human body during and after drug delivery process. In this sense, their lixiviation rate in physiological fluids is a pivotal parameter that should be considered in order to control the release kinetics and the cytotoxicity [120]. Firstly, it is necessary that the nanocarrier is robust enough (chemically stable) to protect the loaded-drugs during their transport to the target tissues or cells. Finally, upon completion of drug release it is desirable that it degrades without causing undesirable accumulation and toxicity in tissues [121].

The MSNs matrix mainly consists of -Si-O- bonds with relatively chemical strong (bond energy of 452 KJ/mol) [122]. However, in aqueous medium they are susceptible to nucleophilic attack by hydroxide of water into the SiO_2_ network. This reaction provokes an hydrolytic breakdown of the siloxane (Si-O-Si) group, leaching orthosilicic acid (Si(OH)_4_), which is biocompatible and well-excreted by urine [123,124,125]. Therefore, a deep comprehension of MSNs solubility and biodegradability is of foremost relevance to ensure their biocompatibility and efficacy in the characteristic conditions of the disease to be treated.

**Figure 6:**
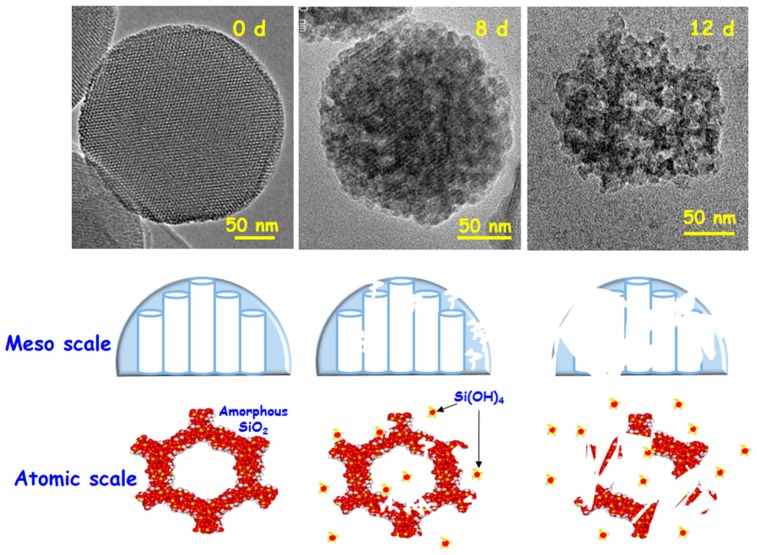
Representative illustration showing the in vitro degradation process in PBS for MSNs. TEM images before and after 8 and 12 days of in vitro assay are shown. The cartoons display the degradation process at the meso and atomic scales.

Figure 6 illustrates the in vitro degradation process of MSNs in phosphate buffer saline (PBS) at 37 °C at different time periods. TEM images indicate that both the structural and morphological features of the nanoparticles are preserved after 8 days of assay. However, after 12 days of test a noticeable alteration of such parameters is clearly observed. These findings reveal that the intrinsic characteristics of MSNs keep stable for enough time to guarantee their functionality as drug delivery systems. Figure 6 also schematizes the degradation process at the meso and atomic scales, representing both the damage in the structural mesopore arrangement (meso scale) as a consequence of the Si(OH)_4_ lixiviation from the silica matrix (atomic scale) to the physiological environment.

**Figure 7:**
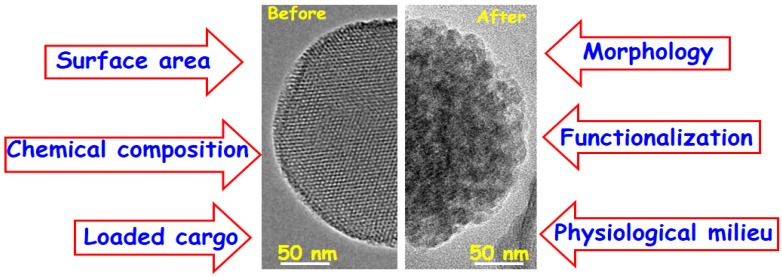
Main factors that drive the degradation of MSNs in physiological environment. TEM images of MSNs before and after being soaked in PBS under physiological conditions reveal the permanence of the structural and morphological characteristics after 8 days of in vitro test.

The main factors that govern the mesoporous silica degradation in physiological environments are schematically summarized in Figure 7.

The effect of MSNs size has been in vitro evaluated by different research groups [126,127], proving that the degradation process did not depend on this parameter. However, there are other parameters such as the morphology, surface area, chemical composition, surface functionalization, loaded cargo and physiological environment, which orchestrate in vitro degradation of MSNs.

Concerning nanoparticle morphology, the lixiviation behavior of spherical- and rod-shaped nanoparticles has been studies, showing faster dissolution rates in the former due to their relative larger outer surface are [128]. Besides, the surface area plays a pivotal role in the degradation rates of MSNs. Thus, the higher the surface area the faster the silica matrix lixiviation due to the greater contact with the physiological medium [129]. Another important parameter that strongly influences in vitro degradation of MSNs is the chemical composition.

Several authors have demonstrated that the doping with different cations, such as Ca(II) and Fe(III) [130,131] increases the dissolution rate of nanoparticles due to the decrease in the silica network connectivity. On the other hand, the design of MSNs in the SiO_2_-ZrO_2_ binary system permits to slow down the dissolution kinetics compared to pure silica MSNs [132]. This finding can be attributed to the fact that the dissolved silica in the medium does not reach the saturation level because of the SiO_2_ recondensation on local zirconium nuclei in the matrix [132]. Regarding to the effect of functionalization on MSNs degradation, different studies have been carried out. Among the different tested organic groups, including phenyl, chloropropyl, aminopropyl-functionalization, PEG-coating [133] or grafting of other polymers [111], PEGylation significantly reduces the dissolution rate. Thus, whereas pure silica MSNs are dissolved from the outside surface towards the inside, PEGylated MSNs start to dissolve oppositely, i.e., from the inside towards the external surface. In addition, the molecular weight of the grafted PEG also affects MSNs degradation, in such a way that longer polymer chains decrease the dissolution rate [133]. Very recently, it has been reported that the loaded-cargo also affects in vitro degradation behavior of MSNs [121]. In this work MSNs were loaded with doxorubicin and matrix dissolution in PBS at 37 °C was monitored vs. time. The obtained results showed a highest degradation rate of drug-loaded nanoparticles due to PBS acidification. However, further studies are needed to evaluate the effect of the chemical nature of the loaded cargo on the lixiviation behavior of MSNs. Additionally, lixiviation behaviors of MSNs were tested in various aqueous media, e.g., PBS, simulated body fluid (SBF), simulated lung fluid (SLF) and simulated gastric fluid (SGF). The obtained results showed the fastest degradation rate in SLF, with comparable behavior in PBS and SBF, and the slowest dissolution in SGF [126]. On the other hand, the degradation process of MSNs has been tested in the presence of proteins by using fetal bovine serum (FBS) [128], demonstrating a decrease in the stability of the nanoparticles. All these studies prove that it is essential a fine tuning the properties of MSNs depending on their biomedical application, because they will be in contact with diverse physiological milieu in the presence of proteins, diverse pH, ionic strength, etc.

Evaluating the in vivo dissolution of MSNs is essential to test their potential clinical translation. In this sense, the biodistribution, biodegradation and excretion of MSNs with different sizes coated or not with PEG have been investigated [134]. The effect of the concentration of degradation products of MSNs in urine after different times following tail vein injection in mice was evaluated. Smaller nanoparticles exhibited significantly lower amount of degradation products and, for a given size, PEGylation slowed down the degradation process. These findings could be ascribed to an easier capture by liver and spleen of bigger MSNs, whereas the PEG coating would decrease their accumulation into these organs by increasing their bloodstream circulation time.

## 4. Benefits and Downsides of MSNs for Drug Delivery

The impact of nanoparticles for drug delivery technologies on the Pharmaceutical Research and Development industry is evident with the creation of more than 2000 start-ups focused on nanomedicine in the last few years [135]. In fact, there are more than 250 nanomedicine products already commercialized or in clinical trials [136]. If we attend to the budgets, the sector of nanopharmaceuticals now represents about the 15% of the total pharmaceutical market, and it is estimated to be worth about $400 billion by 2019 [137].

### 4.1. Benefits of MSNs for Drug Delivery

The reasons for the interest on nanoparticles and, specifically, on MSNs for drug delivery relays on different factors. First of all, and this is probably the most straightforward advantage, is the scale of MSNs interaction with living systems. It is well known that human cells present a broad size variability, but most of them are normally within the micrometer scale. That scale similarity between MSNs and cells would present an intimate interaction. As it has been commented throughout this review, MSNs allow a fine control over the pharmacokinetic profile of the transported therapeutic agent. This is important because when a free drug is administered, independently if it is orally or intravenously delivered, there would be a maximum in the concentration of that drug in the serum. Afterwards, that concentration would be reduced until the next drug administration, which would produce again a concentration peak. This procedure would lead to a “roller coaster” concentration profile, and sometimes the maxima could be over the toxicity levels, and the minima could be under the optimal levels. The use of MSNs as controlled drug delivery systems could keep the drug concentration at optimal levels over prolonged periods of time, improving the efficiency of the treatment and avoiding any type of potential toxicity and the subsequent side effects. MSNs employed for drug delivery can also protect the therapeutic agents during their journey within the body. In this sense, any potential degradation of the cargo would be avoided, which is of special interest when delivering soft therapeutic agents, such as RNAs or proteins. The great loading capacity of MSNs allows transporting two or more drugs into the same nanoparticles, which allows designing combined therapies for tackling multiresistant tumors. Additionally, this feature allows including certain contrast agents for biomedical imaging, which could be very interesting for following up in real time the treatment evolution. Another benefit of using MSNs as drug delivery systems arises from the possibility of providing them with a responsive behavior. This allows a precise control on cargo release upon the use of a stimulus, whether is internal and characteristic of the treated pathology, or externally applied by the clinician. In this sense, the control provided by the stimuli-responsive MSNs delivery system avoids the premature release of the therapeutic agent, which might be of importance when systemic toxicity should be prevented.

### 4.2. Downsides of MSNs for Drug Delivery

It is quite obvious that any nanomedicine able to reach the Clinic would undoubtedly contribute to the benefit of society. However, before reaching the Market, all nanomedicines must succeed in an industrial technology transfer and, obviously, in clinical translation. Regarding the first task, the industrial technology transfer would depend on the scaling up process [138], which together with the reproducibility and the total costs, constitute the ordinary barriers for commercialization. In this sense, the scaling up of the synthesis of MSNs is not trivial, because there are many different factors to take into account during the synthetic procedure. MSNs are often developed in the lab, where milligrams or grams of product are obtained, but the production of large-scale batches under Good Manufacturing Practices (GMP) conditions, which are needed for preclinical screening, clinical trials and clinical use, is a roadblock for their commercialization. Additionally, reproducibility on the synthesis of MSNs at small scale is relatively easy, but at the larger and industrial scale is very difficult to control from batch to batch. In general, and for all nanomedicines, reproducibility is a complicated and expensive process that takes very long to sort out. For all those reasons, the clinical translation of MSNs, in which the therapeutic efficacy alone is not enough, has taken longer than initially desired by researchers in this area. The translation of MSNs to the clinic is somehow stuck in the first milestone that any nanomedicine development should quickly overtake: Are the nanoparticles stable, with great loading capacity, reproducible and scalable? Do far, researchers have answered all the questions but not the last, although advances are taking place quickly in this area.

The next question that researchers should ask themselves from the biological point of view should be regarding their potential toxicity and immunogenicity, which has been found to strongly depend on their surface functionalization. In any case, MSNs have been observed in different animal models to be perfectly biocompatible in which toxicity has been discarded. However, there is still much to do to clearly show the benefits of using this new platform, including in vivo efficacy through pharmacokinetics and pharmacodynamics studies. Additionally, if the MSNs investigated are aimed to be used for cancer treatment, biodistribution should be deeply evaluated, since it is necessary to show that MSNs reach preferentially the tumor tissue.

Once those preclinical studies might be successfully carried out, then the clinical trials for MSNs should be designed. So far, there are not MSNs being evaluated into any clinical trial. This would be a very delicate step, since many nanomedicines fail the clinical translation even before the clinical trials because of reiterative pitfalls. In this sense, from all the nanomedicine scientific papers focused on oncology, only 2% advance to clinical trials. In this sense, the lack of efficiency in humans is a common factor in most nanomedicines that might had showed some kind of success in animal models. The reason for that recurrent failure lies beneath the different physiology of small animals and humans. When dealing with nanomedicines targeting cancer, this is of capital importance, since there are many scientific publications that use xenografts models of human cancer cells in mice to test the nanomedicines. Although it was ok in the past, nowadays it is a very controversial approach and, perhaps, one of the main reasons of the limited clinical success of currently investigated nanomedicines.

A possible alternative could be based on showing great toxicology results in animals and then go straightforward to phase 1 clinical trials, avoiding the preclinical xenograft experiments, which might represent a very expensive and time consuming option. However, toxicity in humans should be carefully approached, since it is one of the main reasons for the pharmaceutical companies to exclude a nanotherapeutic during the different stages of clinical trials. In this sense, the nanocarriers analyzed should be non-toxic themselves and they should be excreted completely from the body via the liver and/or the kidneys. Regarding this, safety and renal or hepatic clearance must be some of the basic criteria when considering the clinical translation of nanoparticles for drug delivery [139].

Another key point during the planning of the clinical trials should be dealing with the regulation agencies, such as the American Food and Drug Administration (FDA), or the European Medicines Agency (EMA). Nowadays there is a lack of specific requirements for nanomedicines from those agencies, and the evaluation process follows the same path as for small-molecules drugs. This means that every novel nanocarrier for drug delivery has to follow a complete evaluation process, even if the transported drug (Active Pharmaceutical Ingredient) alone has been already accepted for clinical use. This supposes a bottleneck for the translation of novel nanomedicines, with only 5% of the initial nanomedicines initially evaluated succeeding in the market authorization. It is expected that in the near future the regulatory agencies might develop specific requirements for nanomedicines to accelerate the translation from the lab to the clinic.

Last, but not least, it is also very important that the healthcare professionals might understand the potential benefits of using novel nanomedicines. The Pharmaceutical Industry should clearly demonstrate the therapeutic efficacy, so clinicians, who also demand easy and safe administration routes, would use them in the near future.

## Figures and Tables

**Table 1:** Different active targeting strategies for MSNs.

**Targeting Ligand ^a^**	**Tumor Cell Receptor ^b^**	**Target Cell Line ^c^**	**Ref.**
Tf	TfR	PANC-1, BT-549	[34]
Tf	TfR	HeLa	[35]
Tf	TfR	HT1080	[36]
EGF	EGFR	HuH-7	[37]
FA	FAR (FR-α)	Hela, PANC, U2Os, MDA-MB-231, SK-BR-3, MiaPaca-2, LnCAP	[38,39,40,41,42,43,44,45,46]
Methotrexate	FR-α	HeLa	[47]
Anisamide	Sigma receptor	ASPC-1	[44]
TAT peptides	Importing α and β receptors	Hela; MCF-7/ADR	[48,49,50]
IL-13 peptide	IL-13Rα2	U251	[51]
Anti-herceptin	HER2	SK-BR3	[52]
Anti-HER2/neu	HER2/neu	BT474	[53]
Anti-ErbB2	ErbB2	MCF-7	[54]
Anti-ME1	Mesothelin	MM	[55]
Anti-TRC105	CD105/endoglin	HUVECs	[56]
MABG	NET	NB1691-luc	[57]
RGD-type peptide (RDGRC)	NRP-1	HOS	[58]
ConA	SA	HOS	[59]
HA	CD44	MCF-7, MDA-MB-231, 4T1	[60]
**Targeting Ligand ^a^**	**Tumor Blood Vessel Receptor ^b^**	**Target Cell Line ^c^**	**Ref.**
c(RGDyK)	α_ν_β_3_ integrins	U87-MG	[61]
cRGD	α_ν_β_3_ integrins	MDA-MB 435	[34]
K7RGD; c-RGDFK	α_ν_β_3_ integrins	HeLa	[62]
K_8_(RGD)_2_	α_ν_β_3_ integrins	U87-MG	[63]
N_3_GPLGRGRGDK-Ad	α_ν_β_3_ integrins	SCC-7, HT-29	[61]
N_3_RGDFFFFC	α_ν_β_3_ integrins	U87-MG	[64]
Thiolated-RGD	α_ν_β_3_ integrins	A375, HepG2, MCF-7, Neuro-2a	[66]
Anti-(VCAM-1)	(VCAM-1)R	HUVEC-CS	[67]
VEGF	VEGFR	U87-MG	[68]

^a^ Tf: Transferrin; FA: Folic acid; EGFR: Epidermal growth factor; TAT: Transactivator of transcription; IL-13: Interleukin-13; MABG: metaaminobenzyl guanidine (meta-iodobenzylguanidine analogue); ConA: concanavalin A; c(RGD): Cyclic RGD (Arg-Gly-Asp); c(RGDyK): Cyclo(Arg-Gly-Asp-d-Phe-Lys); K_7_RGD: linear RGD peptide sequence with 7 consecutive lysine residues; K_8_(RGD)_2_ cationic peptide containing 2 RGD sequences; VCAM-1: vascular cell adhesion molecule 1; VEGFR: Vascular endothelial growth factor; ^b^ TfR: transferrin receptor; EGFR: epidermal growth factor receptor; FAR (FR-α): Folic acid receptor; IL-13Rα2: interleukin-13 receptor subunit alpha-2; HER2: epidermal growth factor receptor; ErbB2: Receptor tyrosine-protein kinase 2; NET: norepinephrine transporter; NRP-1: neuropilin receptors; SA: sialic acid; (VCAM-1)R: vascular cell adhesion molecule 1 receptor; VEGFR: Vascular endothelial growth factor receptor; ^c^ PANC-1: human pancreatic carcinoma, epithelial-like cell line; BT-549: human breast carcinoma cell line; HeLa: Human epithelial cells from a fatal cervical carcinoma; HT1080: Fibrosarcoma cell line; HuH-7: Human hepatoma cell line; U20S: Human osteosarcoma cell line; MDA-MB 231 and 435: Human breast carcinoma cell lines; SK-BR-3: Human breast adenocarcinoma cell line; MiaPaca-2: Human pancreatic carcinoma cell line; LnCAP: human prostate cancer cell line; ASPC-1: Human pancreas adenocarcinoma cell line; MCF-7/ADR: (ADR)-selected human breast cancer cell line; U251: glioma cell line; BT474: Human breast cancer cell line; MM: Multiple myeloma cell line; HUVEC: human umbilical vein endothelial cell line; NB1691-luc: human neuroblastoma cells; HOS: human osteosarcoma cell line; MDA-MB-23: human breast cancer cell line; 4T1: mouse breast cancer cell line; U87-MG: human primary glioblastoma cell line; SCC-7: Squamous cell carcinoma; HT-29: human intestinal epithelial cells; A375: Human amelanotic melanoma cell line; HepG2: human hepatoblastoma-derived cell line; Neuro-2a: Mouse neuroblastoma cell line; HUVEC: human umbilical vein endothelial cell line.

**Table 2:** Internal stimuli-responsive strategies for smart drug delivery MSNs.

Stimulus	Responsive Linker	Blocking Cap	Ref.
pH	Acetal linker	Au NPs	[78]
pH	Boronate ester	Fe_3_O_4_ NPs	[79]
pH	Ferrocenyl moieties	β-CD-modified CeO_2_ NPs	[80]
pH	PAH-PSS PEM	PAH-PSS PEM	[81]
pH	Aromatic amines	CDs	[82]
pH	Benzoic-imine bonds	Polypseudorotaxanes	[83]
pH	CaP soluble at acid pH	CaP coating	[84]
pH	Self-immolative polymer	Self-immolative polymer	[85]
pH	Gelatin	Gelatin coating	[45,86]
pH	3,9-Bis(3-aminopropyl)-2,4,8,10-tetraoxaspiro [5.5] undecane (ATU)	Poly(acrylic acid) PAA	[59]
Redox potential	—S—S—	ssDNA	[87]
Redox potential	—S—S—	PEG	[88]
Redox potential	—S—S—	CdS NPs	[89]
Redox potential	—S—S—	PPI dendrimer	[90]
Enzymes	MMP-degradable gelatin	Gelatin coating	[91]
Enzymes	β-galactosidase-cleavable oligosaccharide	β-galacto-oligosaccharide	[92]
Enzymes	MMP9-sensitive peptide sequence (RSWMGLP)	Avidin	[93]
Enzymes	Protease-sensitive peptide sequences (CGPQGIWGQGCR)	PNIPAm-PEGDA shell	[94]
Enzymes	α-amylase and lipase cleavable stalks	CDs	[95]
Enzymes	HRP-polymer nanocapsule	-	[96]
Enzymes	Phosphate-phosphate APasa-hydrolizable bonds	ATP	[97]
Small molecules	Ionizable benzimidazole group	CD-modified glucose oxidase	[98]
Small molecules	pAb	pAb	[99]
Small molecules	ATP aptamer	ATP aptamer	[100]

PNIPAm: Poly(*N*-isopropylacrylamide); Poly(acrylic acid) PAA ssDNA: single-stranded DNA; CB[6]: Cucurbit[6]uril; PEI: poly(propylene imine); PEG: poly(ethylneglycol); CD: cyclodextrin; PAH: poly (allylamine hydrochloride); PSS: sodium poly(styrene sulfonate); PEM: polyelectrolyte multilayers; APase: acid phosphatase; PEGDA: poly(ethylene glycol) diacrylate; HRP: enzyme horseradish peroxidase; ATP: adenosine triphosphate; pAb: polyclonal antibody; MMP: matrix metalloproteinase.
